# Impact of the COVID-19 pandemic stay at home order and social isolation on physical activity levels and sedentary behavior in Brazilian adults

**DOI:** 10.31744/einstein_journal/2021AE6156

**Published:** 2021-02-25

**Authors:** João Paulo Botero, Breno Quintella Farah, Marilia de Almeida Correia, Mara Cristina Lofrano-Prado, Gabriel Grizzo Cucato, Grace Shumate, Raphael Mendes Ritti-Dias, Wagner Luiz do Prado

**Affiliations:** 1 Universidade Federal de São Paulo SantosSP Brazil Universidade Federal de São Paulo, Santos, SP, Brazil.; 2 Universidade Federal Rural de Pernambuco RecifePE Brazil Universidade Federal Rural de Pernambuco, Recife, PE, Brazil.; 3 Universidade Nove de Julho São PauloSP Brazil Universidade Nove de Julho, São Paulo, SP, Brazil.; 4 São PauloSP Brazil Psicóloga, São Paulo, SP, Brazil.; 5 Northumbria University NewcastleNE United Kingdom Northumbria University, Newcastle, NE, United Kingdom.; 6 California State University San BernardinoCA United States California State University, San Bernardino, CA, United States.

**Keywords:** Coronavirus, Coronavirus infections, COVID-19, Quarantine, Sedentary lifestyle, Sedentary behavior, Physical inactivity

## Abstract

**Objective:**

To investigate the impact of the coronavirus 2019 pandemic on physical activity levels and sedentary behavior among Brazilians residents aged ≥18 years.

**Methods:**

An online survey was distributed through a social media platform between May 5 and 17, 2020. Participants completed a structured questionnaire in Google Forms, which assessed the physical activity level and sedentary behavior of adults in Brazil during the pandemic.

**Results:**

Age (OR: 0.98; 95%CI: 0.97-0.99), chronic disease (OR: 1.29; 95%CI: 1.03-1.63), physical inactivity before the coronavirus 2019 pandemic (OR: 2.20; 95%CI: 1.78-2.72) and overweight (OR: 1.34; 95%CI: 1.09-1.65) showed higher risk of impact on physical activity levels. Increased sitting time was associated with older individuals (OR: 0.97; 95%CI: 0.96-0.98), inactivity (OR: 1.51; 95%CI: 1.16-1.96), chronic disease (OR: 1.65; 95%CI: 1.23-2.22), higher number of days in social isolation (OR: 1.01; 95%CI: 1.00-1.02) and higher schooling levels (OR: 1.87; 95%CI: 1.26-2.78).

**Conclusion:**

Our results demonstrated that advanced age, chronic disease and physical inactivity before social isolation had a greater risk of impact on reduced physical activity levels and increased sitting time during the coronavirus 2019 disease pandemic.

## INTRODUCTION

The novel coronavirus disease 2019 (COVID-19) pandemic is one of the most colossal and far-reaching public health problems in human history. Up to September 10, 2020, almost 27 million cases and approximately 899 thousand deaths had been reported worldwide.^(1)^ Stay at home orders and social distancing are some of the few strategies available to contain the spread of COVID-19. Billions of people are in social isolation around the world, which may lead to unhealthy behaviors, such as decreased physical activity and time spent using electronic devices such as televisions, computers and smartphones.^(2-4)^

The tactics utilized in combating the COVID-19 pandemic have varied from country to country. The strategies have depended on contrasting cultures, political views, social isolation, and available resources among other factors. Brazil has recently become the new epicenter of the COVID-19 pandemic, and the consequences of social isolation on physical activity and sedentary behavior have been poorly addressed.^(5)^

## OBJECTIVE

To investigate the impact of the coronavirus 2019 pandemic on physical activity levels and sedentary behavior among Brazilians residents aged ≥18 years.

## METHODS

### Sample and ethics

This survey was conducted in Brazil between May 5 and 17, 2020. Participants were invited through social media to answer a structured questionnaire. This study was approved by the Ethics Committee of the *Universidade Nove de Julho* (UNINOVE) before data collection (CAAE: 30890220.4.0000.5511, protocol 4.002.943). Participants were anonymous. All procedures follow the national legislation and the Declaration of Helsinki. Inclusion criteria were age ≥18 years and to answer all questions.

### Procedures

After approval by the Ethics Committee, a questionnaire in Google Forms was presented to participants as 70 questions, distributed in seven sections: personal information; COVID-19 personal care; physical activity; eating behavior; habits contributing to health risks; mental health and overall health.^(6)^ The format was developed for senior researchers, who have PhD degree in various subjects (public health, science, nutrition, physiology, human movement science, neuroscience and behavior), to analyze and draw conclusions. The following includes questions used in the present analysis. After filling in the questionnaire, the entire form was redirected to a spreadsheet generated by Google, in Microsoft Excel format, for further analysis. For the purpose of the present study only personal information; physical activity; health risk habits; and overall health domains were analyzed. The details of these domains are described below.

### Instrument

Concerning personal information, information about sex (possible answers: male or female); date of birth (DD/MM/YYYY); type of residence (possible answers: house or apartment); educational level (possible answers: elementary, high school, undergraduate, and graduate) and days of social isolation (open question) were obtained.

To assess physical activity habits, participants were asked: “How often do you exercise a week?” (possible answers: none to 7 days); “How long do you exercise?” (possible answers: none; less than 30 minutes; between 30 and 60 minutes, and more than 60 minutes); “How long have you been engaged in a certain physical activity?” (possible answer: less than 1 month; between 1 to 3 months; between 3 to 6 months; more than 6 months; and I am not exercising); “How intense is the physical activity?” (possible answers: low *– i.e.* bathing, shaving, driving, washing the dishes, making the bed; medium/moderate *– i.e.* gardening, playing volleyball, water aerobics, bike riding, brisk walking; high - *i.e.* stair climbing, swimming, jumping rope, playing soccer, running, and I am not exercising); “What type of exercise do you practice?” (possible answers: walking/jogging; resistance training; core exercise; no exercise and other - open question).

Based on these responses, time spent during each exercise session throughout the study was multiplied by the number of days spent exercising each week. Those that reached 150 minutes or more of moderate to vigorous physical activity were considered, physically active, while those who fell below this threshold were classified as inactive.

Additionally, there were questions related to the impact of COVID-19 on physical activity and sedentary behavior: “How much has the COVID-19 pandemic interfered with your daily physical activity habits?” (possible answers: none, a little, a lot, I do not exercise). For analysis purposes, “none” and “a little” were considered as “no”, and a lot as “yes”. Those who responded “I do not exercise” were excluded from this analysis.

The section about health risk habits included information regarding health care related to social habits, such as smoking, drinking alcohol, screen time on a smartphone, computer or television, and the time the participant spent sitting. The question for this section was: “Due to social isolation, have you spent more time seated?” (possible answers: yes or no).

The section about overall health assesses the presence of diagnosed diseases. From the list of diseases, the participant could mark all that apply (possible answers: hypertension, diabetes, high cholesterol, high triglycerides, depression, arthritis/osteoarthritis/ rheumatism, asthma, cardiopathy, or other). For research purposes, if the participant marked any of these options, they were considered as “yes” for chronic diseases. If none of them were marked, the participant was considered as “no” for chronic disease.

In this section, it was also asked “What is your weight (in kilograms)?” and “What is your height (in centimeters)?” as well as “What is your body mass index (BMI)?” This was calculated by dividing body weight by squared height. Overweight was defined as a BMI ≥25.0kg/m^2^.

### Statistical analysis

All statistical analyses were made in the SPSS/Predictive Analytics Software (PASW) version 20 (IBM Corp, New York, USA). Frequency and mean (95% confidence interval) were used for descriptive analysis.

Multiple logistic regressions were conducted to identify predictors of impact on physical activity levels and increased sitting time during the COVID-19 pandemic. Furthermore, stepwise regression techniques were employed to enter the covariates into the model, using only variables with p<0.20 in the bivariate analyses. In the regressions, only variables with p<0.05 remained in the final model. The Hosmer-Lemeshow test was used to assess the model’s goodness-of-fit and suitability to the set of observations. The significance level was set at p<0.05 for all analyses.

## RESULTS

Out of the 1,895 individuals who were enrolled in the present study, three did not inform their sex and 11 did not report their age. Therefore, the final sample comprised 1,881 individuals (1,103 women). Table 1 shows general characteristics, physical activity levels an sedentary behavior of the sample.

**Table 1:** General characteristics, physical activity level and sedentary behavior of Brazilians isolated during the COVID-19 pandemic

Variables	Values
Female	58.7
Age, years	39±13
Social isolation, days	44±15
Elementary or high school	8.8
Type of housing, apartment	55.2
Chronic diseases	32.0
Overweight	50.6
Physically active	28.7
Impact of the COVID-19 on PA level	61.2
Increased sitting time due the COVID-19	82.3

Results presented as frequency or mean±standard deviation. PA: physical activity; COVID-19: coronavirus disease 2019.

Table 2 shows comparisons of the impact of the COVID-19 pandemic on physical activity (yes *versus* no) and sitting time (yes *versus* no). Participants who changed their physical activity levels during the COVID-19 pandemic were younger (p=0.024), more overweight (p=0.003) and less active before the pandemic (p<0.001). Participants who increased their sitting time during the COVID-19 pandemic were younger (p=0.024), with more days of social isolation (p=0.005), higher prevalence of undergraduate and graduate educational levels (p=0.019), more overweight (p=0.003) and less active before the pandemic (p=0.001).

**Table 2:** Characteristics of participants with and without impact on physical activity levels and increased sitting time due to COVID-19 pandemic

Variables	Impact on PA level		p value	Increased sitting time		p value
	No	Yes		No	Yes	
Age, years	39±13	38±12	0.024	42±12	38±13	<0.001
Social isolation, days	44±15	44±14	0.764	41±14	44±14	0.005
Sex			0.550			0.357
Male	38.0	62.0		16.7	83.3	
Female	39.4	60.6		18.4	81.6	
Schooling level			0.401			0.019
Elementary or high school	42.1	57.9		24.4	75.6	
Undergraduate or graduate	38.5	61.5		17.0	83.0	
Chronic diseases			0.057			0.039
Yes	35.5	64.5		15.0	85.0	
No	40.4	59.6		18.9	81.1	
Overweight			0.003			0.993
Yes	35.4	64.6		17.6	82.4	
No	42.3	57.7		17.4	82.6	
Physically active			<0.001			0.001
Yes	52.3	47.7		22.1	77.9	
No	32.8	67.3		15.9	84.1	

PA: physical activity.

Table 3 shows the predictors of impact on physical activity levels and increased sitting time due to the COVID-19 pandemic. Adjusted analysis shows adults who were older (p=0.001), inactive (p<0.001), overweight (0.005) and suffering from chronic diseases (0.027) had a greater impact on physical activity levels due to COVID-19. Increased sitting time was associated with older (p<0.001), inactive (p=0.002), presence of chronic diseases (p=0.001), more days in social isolation (p=0.002) and high schooling level (p=0.002).

**Table 3:** Multiple logistic regression models predicting impact on impact on physical activity levels and increased sitting time due to COVID-19

Variables	β (EP)	OR	95%CI	p value
Impact on PA level^*^				
Age, years	-0.013 (0.004)	0.98	0.97-0.99	0.001
Physically active, yes as reference	0.790 (0.108)	2.20	1.78-2.72	<0.001
Chronic diseases, no s reference	0.259 (0.117)	1.29	1.03-1.63	0.027
Overweight, no s reference	0.297 (0.105)	1.34	1.09-1.65	0.005
Impact on sitting time^†^				
Age, years old	-0.029 (0.005)	0.97	0.96-0.98	<0.001
Physically active, yes s reference	0.414 (0.134)	1.51	1.16-1.96	0.002
Chronic diseases, no s reference	0.504 (0.150)	1.65	1.23-2.22	0.001
Social isolation, days	0.013 (0.004)	1.01	1.00-1.02	0.002
Schooling, low^‡^ as reference	0.629 (0.201)	1.87	1.26-2.78	0.002

* Hosmer-Lemeshow test: χ^2^ 11.231, p=0.189; ^†^ Hosmer-Lemeshow test: χ^2^ 6.825, p=0.556; ^‡^ elementary or high school. β (EP): regression coefficient (error-standard); OR: odds ratio; 95%CI: 95% confidence interval; PA: physical activity.

## DISCUSSION

The results of this study indicated that social isolation imposed by the COVID-19 pandemic led to a reduction in physical activity levels and an increase in sedentary behavior in Brazilian adults. Age, presence of chronic disease, physical inactivity, and excessive weight before the COVID-19 pandemic induce greater risk of impact on physical activity levels. Increased sitting time is associated with age, previous physical inactivity, presence of chronic diseases, more days in social isolation and higher schooling levels.

In this study, more than 50% of men and women reported that the COVID-19 pandemic reduced their physical activity levels. Interestingly, these alterations occurred similarly in all age groups. These results are consistent with the reports of a wearable physical activity tracking device used by more than 30 million people worldwide. It identified a 7% to 38% reduction in step count during the week of March 15, 2020, when compared with the same period in the previous year.^(7)^

This data raises concern since both physical inactivity and longer sitting time are independent predictors of all-cause and cardiovascular mortality. The adverse effects of the variables physical inactivity and sedentary behavior are multiple and directly related to sarcopenia,^(8)^ increased falls in elderly people,^(9,10)^ hypertension,^(11)^ insulin resistance and type II diabetes,^(12)^ obesity,^(13)^ cancer^(14)^ and ultimately higher mortality rate.^(15,16)^

Physical inactivity is the fourth leading cause of death worldwide, and it is also associated with reduced life expectancy and quality of life.^(17,18)^ Of note, physical inactivity related-costs, such as health expenses and productivity losses, exceeded US$ 67.5 billion dollars, in 2013.^(19)^

The deterioration of well-being and quality of life caused by inactivity and increased sedentary behavior emphasizes the fundamental importance of physical activity in the life of every individual. Increasing physical activity, such as the number of steps, significantly improves health and it has been inversely associated with developing chronic diseases.^(17-20)^ Guidelines for increase physical activity^(21)^ are crucial in preserving muscle mass and neuromuscular function, cardiorespiratory fitness and glucose metabolism, especially when unexpected circumstances (such as the recent outbreak of COVID-19) cause a strong restriction of daily movement in comparison to normal life. Of particular importance, individuals who were older, had chronic diseases, or were inactive before the pandemic showed an even greater decrease in physical activity levels and an increase in sitting time.^(22)^

Interestingly, we demonstrated that older adults, who have some chronic disease and were already inactive, were those who suffered more during the pandemic, increasing sitting time and reducing physical activity levels. They also should probably be the last to leave social isolation. These people are precisely those who most need physical activity, especially during personal isolation. Our findings prompt the need to implement preventative public health measures to support the practice of physical activities at home during the pandemic. This is especially imperative because the termination of these arduous circumstances is still unknown. Several studies have suggested that home-based training during the pandemic can be effective, although more evidence is needed to determine long term health benefits and consequences.^(23-26)^ Use of social media or other online resources specifically geared toward physical activity may be a viable method in helping increase the practice of regular physical activities at home.

This study has limitations that are worth highlighting. The cross-sectional design of this study is an evident limitation because no causality can be inferred. Additionally, online surveys are susceptible to information and social biases, especially without direct measures of physical activity and sedentary behavior. On the other hand, the large sample size and its timely assessment of health behaviors make our findings important and quite relevant.

## CONCLUSION

Our study clearly demonstrated that age, chronic disease and physical inactivity before social isolation had an immense impact on physical activity levels, as well as a detrimental influence on increased sedentary behavior during the coronavirus disease 2019 pandemic.
