# Supplementary material for: Impact of the COVID-19 pandemic stay at home order and social isolation on physical activity levels and sedentary behavior in Brazilian adults
Source: Einstein (Sao Paulo). 2021 Feb 25;19:eAE6156. doi: 10.31744/einstein_journal/2021AE6156 (PMC7909004; doi:10.31744/einstein_journal/2021AE6156)
Supplement: Translation

# Translation: Impacto da permanência em casa e do isolamento social, em função da COVID-19, sobre o nível de atividade física e o comportamento sedentário em adultos brasileiros

**Authors:** João Paulo Botero, Breno Quintella Farah, Marilia de Almeida Correia, Mara Cristina Lofrano-Prado, Gabriel Grizzo Cucato, Grace Shumate, Raphael Mendes Ritti-Dias, Wagner Luiz do Prado

**Affiliations:** 1 Brazil Universidade Federal de São Paulo, Santos, SP, Brazil.; 2 Brazil Universidade Federal Rural de Pernambuco, Recife, PE, Brazil.; 3 Brazil Universidade Nove de Julho, São Paulo, SP, Brazil.; 4 Brazil Psicóloga, São Paulo, SP, Brazil.; 5 United Kingdom Northumbria University, Newcastle, NE, United Kingdom.; 6 United States California State University, San Bernardino, CA, United States.

**Keywords:** Coronavírus, Infecções por coronavírus, COVID-19, Quarentena, Sedentarismo, Comportamento sedentário, Inatividade física

## Abstract

**Objetivo:**

Investigar o impacto da pandemia de coronavírus 2019 sobre o nível de atividade física e comportamento sedentário entre brasileiros com idade ≥18 anos.

**Métodos:**

Uma pesquisa on-line foi distribuída por meio de mídias sociais entre 5 e 17 de maio de 2020. Os participantes responderam a um questionário estruturado no *Google Forms*, que avaliou o nível de atividade física e comportamento sedentário de adultos no Brasil durante a pandemia.

**Resultados:**

Idade (RC: 0,98; IC95%: 0,97-0,99), doença crônica (RC: 1,29; IC95%: 1,03-1,63), inatividade física antes da pandemia de coronavírus 2019 (RC: 2,20; IC95%: 1,78-2,72) e excesso de peso (RC: 1,34; IC95%: 1,09-1,65) apresentaram maior risco de impacto no nível de atividade física. O aumento do tempo sentado foi associado a indivíduos mais velhos (RC: 0,97; IC95%: 0,96-0,98), inatividade (RC: 1,51; IC95%: 1,16-1,96), doença crônica (RC: 1,65; IC95%: 1,23-2,22), maior número de dias no isolamento social (RC: 1,01; IC95%: 1,00-1,02) e níveis de escolaridade mais altos (RC: 1,87; IC95%: 1,26-2,78).

**Conclusão:**

Nossos resultados demonstraram que a idade, a presença de doenças crônicas e a inatividade física antes do isolamento social tiveram maior risco de impacto na redução do nível de atividade física e maior tempo sentado durante a pandemia de coronavírus 2019.

## INTRODUÇÃO

A pandemia da doença causada pelo novo coronavírus 2019 (COVID-19) é um dos problemas de saúde pública mais colossais e abrangentes da história da humanidade. Até 10 de setembro de 2020, foram notificados quase 27 milhões de casos e aproximadamente 899 mil óbitos no mundo.^(1)^ As recomendações de permanência em casa e distanciamento social são algumas das poucas estratégias disponíveis para conter a disseminação da COVID-19. Bilhões de pessoas estão em isolamento social no mundo inteiro, o que pode levar a comportamentos pouco saudáveis, como a redução do nível de atividade física e o aumento do tempo gasto com dispositivos eletrônicos, como televisores, computadores e *smartphones*.^(2-4)^

As táticas utilizadas no combate à pandemia da COVID-19 têm variado em diversos países. As estratégias dependem, entre outros fatores, de contrastes culturais, visões políticas, isolamento social e recursos disponíveis. Recentemente, o Brasil se tornou o novo epicentro da pandemia da COVID-19, e as consequências do isolamento social sobre atividade física e o comportamento sedentário não estão sendo tratadas de forma adequada.^(5)^

## OBJETIVO

Investigar o impacto da pandemia de coronavírus 2019 sobre o nível de atividade física e comportamento sedentário entre brasileiros com idade ≥18 anos.

## MÉTODOS

### Amostra e aspectos éticos

Este levantamento foi conduzido no Brasil entre 5 e 17 de maio de 2020. Os participantes foram convidados, por meio das mídias sociais, a responder um questionário estruturado. Antes da coleta de dados, o estudo foi aprovado pelo Comitê de Ética da Universidade Nove de Julho (UNINOVE) (CAAE: 30890220.4.0000.5511, parecer: 4.002.943). Os participantes permaneceram anônimos. Todos os procedimentos foram realizados de acordo com a legislação nacional e a Declaração de Helsinque. Os critérios de inclusão foram ter pelo menos 18 anos de idade e responder a todas as perguntas.

### Procedimentos

Após a aprovação pelo Comitê de Ética, os participantes receberam um questionário no *Google Forms* contendo 70 perguntas distribuídas, em sete seções: informações pessoais; cuidados pessoais contra a COVID-19; atividade física; comportamento alimentar; hábitos de risco para a saúde; saúde mental e saúde geral.^(6)^ O formato foi desenvolvido para que pesquisadores seniores, com Doutorado em várias áreas (saúde pública, ciência, nutrição, fisiologia, ciência do movimento humano, neurociência e comportamento) pudessem analisar e tirar conclusões. Algumas perguntas usadas na presente análise são apresentadas a seguir. Após o preenchimento do questionário, o formulário foi redirecionado para uma planilha gerada pelo *Google*, em formato Microsoft Excel, para análise posterior. Para fins do presente estudo, foram analisados somente os seguintes domínios: informações pessoais; atividade física; hábitos de risco para a saúde e saúde geral. Os detalhes desses domínios também são descritos a seguir.

### Questionário

Em relação às informações pessoais, foram obtidos dados sobre sexo (respostas possíveis: masculino ou feminino); data de nascimento (no formato DD/MM/AAAA); tipo de residência (respostas possíveis: casa ou apartamento); escolaridade (respostas possíveis: ensino fundamental, ensino médio, graduação e pós-graduação) e número de dias de isolamento social (pergunta aberta).

Para avaliar os hábitos de atividade física, foram feitas as seguintes perguntas aos participantes: “Atualmente quantas vezes por semana você se realiza atividade física?” (respostas possíveis: nenhuma a sete vezes); “Qual a duração de cada sessão de atividade física?” (respostas possíveis: não faço exercício físico; menos de 30 minutos; entre 30 e 60 minutos; mais de 60 minutos); “Há quanto tempo você pratica essa atividade física?” (respostas possíveis: menos de 1 mês; entre 1 e 3 meses; entre 3 e 6 meses; mais de 6 meses; e não estou me exercitando); “Qual a intensidade dessa atividade física?” (respostas possíveis: não pratico atividade física; leve, e foram dados os exemplos: tomar banho, fazer a barba, dirigir, lavar a louça, arrumar a cama; moderada, e foram dados os exemplos: jardinagem, jogar vôlei, hidroginástica, pedalar, caminhar rápido; intensa/vigorosa, e foram dados os exemplos: subir escada, natação, pular corda, jogar futebol, corrida); “Atualmente que tipo de exercício físico o(a) senhor(a) realiza?” (respostas possíveis: caminhada; musculação; exercícios funcionais; não realizo atividade física; e outros –pergunta aberta).

Com base nas respostas, o tempo gasto durante cada sessão de atividade física durante o estudo foi multiplicado pela quantidade de dias da semana em que o participante se exercitou. Os participantes que somaram 150 minutos ou mais de atividade física de intensidade moderada a alta foram classificados como fisicamente ativos, enquanto aqueles que não atingiram esse limiar foram classificados como inativos.

Além disso, havia perguntas relacionadas ao impacto da COVID-19 na atividade física e no comportamento sedentário: “O quanto a pandemia da COVID-19 interferiu em sua rotina de atividade física?” (respostas possíveis: nada, um pouco, muito e não realizo atividade física). Para fins da análise, as opções “nada” e “um pouco” foram consideradas como “não”, e a opção “muito” foi considerada como “sim”. Os participantes que responderam “não realizo atividade física” foram excluídos desta análise.

Os hábitos de risco para a saúde incluíram informações sobre cuidados com a saúde relacionadas a hábitos sociais, como tabagismo, consumo de bebidas alcoólicas, tempo de tela em dispositivos, como *smartphone*, computador ou televisão, e o tempo que o participante passou sentado. A pergunta dessa seção foi: “Devido ao isolamento social, o senhor(a) passa mais tempo sentado(a)?” (respostas possíveis: sim ou não).

Na seção sobre saúde geral, foi avaliada a presença de doenças diagnosticadas. O participante tinha que marcar todas as opções aplicáveis em uma lista de doenças (respostas possíveis: hipertensão; diabetes; colesterol alto; triglicérides alto; depressão; artrite/osteoartrite/reumatismo; asma; cardiopatia; outras ou nenhuma). Para fins da pesquisa, se o participante assinalasse alguma dessas opções, a resposta seria considerada como “sim” para presença de doenças crônicas. Se nenhuma das opções fosse assinalada, a resposta seria considerada como “não” para presença de doenças crônicas.

Nessa seção, o participante também teve que responder às seguintes perguntas: “Qual seu peso atualmente (em quilogramas)?”; “Qual sua altura atualmente (em centímetros)?” e “Qual o seu índice de massa corporal (IMC)?”. A partir disso, o IMC foi calculado dividindo o peso corporal pela altura ao quadrado. Sobrepeso foi definido como IMC ≥25,0kg/m^2^.

### Análise estatística

Todas as análises estatísticas foram feitas utilizando o software SPSS/*Predictive Analytics Software* (PASW), versão 20 (IBM Corp, Nova York, EUA). Para a análise descritiva, foram usadas a frequência e a média (intervalo de confiança de 95%).

Foram feitas análises de regressão logística múltipla para identificar os preditores do impacto sobre o nível de atividade física e o aumento do tempo sentado durante a pandemia de COVID-19. Além disso, foram aplicadas técnicas de regressão passo a passo para inserir as covariáveis no modelo, usando apenas variáveis com p<0,20 nas análises bivariadas. Nas regressões, somente as variáveis com p<0,05 foram mantidas no modelo final. O teste de Hosmer-Lemeshow foi usado para avaliar a qualidade do ajuste e a adequação ao conjunto de observações do modelo. Para todas as análises, foi estabelecido um nível de significância de p<0,05.

## RESULTADOS

Dos 1.895 indivíduos incluídos neste estudo, três não informaram o sexo e 11 não relataram a idade. Desse modo, a amostra final foi composta de 1.881 indivíduos (1.103 do sexo feminino). As características gerais, o nível de atividade física e o comportamento sedentário da amostra incluida neste estudo estão descritos na tabela 1.

**Tabela 1:** Características gerais, o nível de atividade física e comportamento sedentário de brasileiros isolados durante a pandemia da COVID-19

Variáveis	Valores
Sexo feminino	58,7
Idade, anos	39±13
Isolamento social, dias	44±15
Ensino fundamental ou ensino médio	8,8
Tipo de residência, apartamento	55,2
Doenças crônicas	32,0
Sobrepeso	50,6
Fisicamente ativo	28,7
Impacto da COVID-19 sobre o nível de AF	61,2
Aumento do tempo sentado devido à COVID-19	82,3

Resultados apresentados como frequência ou média±desvio padrão. AF: atividade física; COVID-19: doença novo coronavírus 2019.

A tabela 2 apresenta as comparações do impacto da pandemia da COVID-19 sobre a atividade física (sim *versus* não) e o tempo sentado (sim *versus* não). Os participantes que mudaram seu nível de atividade física durante a pandemia da COVID-19 eram mais jovens (p=0,024), apresentavam mais excesso de peso (p=0,003) e eram menos ativos antes da pandemia (p<0,001). Os participantes que tiveram aumento do tempo sentado durante a pandemia da COVID-19 eram mais jovens (p=0,024) e apresentavam mais dias de isolamento social (p=0,005), maior prevalência de graduação e pós-graduação como nível de escolaridade (p=0,019), mais sobrepeso (p=0,003) e eram menos ativos antes da pandemia (p=0,001).

**Tabela 2:** Características dos participantes com e sem impacto sobre o nível de atividade física e o aumento do tempo sentado devido à pandemia da COVID-19

Variáveis	Impacto no nível de AF		Valor de p	Aumento do tempo sentado		Valor de p
	Não	Sim		Não	Sim	
Idade, anos	39±13	38±12	0,024	42±12	38±13	<0,001
Isolamento social, dias	44±15	44±14	0,764	41±14	44±14	0,005
Sexo			0,550			0,357
Masculino	38,0	62,0		16,7	83,3	
Feminino	39,4	60,6		18,4	81,6	
Escolaridade			0,401			0,019
Ensino fundamental ou ensino médio	42,1	57,9		24,4	75,6	
Graduação ou pós-graduação	38,5	61,5		17,0	83,0	
Doenças crônicas			0,057			0,039
Sim	35,5	64,5		15,0	85,0	
Não	40,4	59,6		18,9	81,1	
Sobrepeso			0,003			0,993
Sim	35,4	64,6		17,6	82,4	
Não	42,3	57,7		17,4	82,6	
Fisicamente ativo			<0,001			0,001
Sim	52,3	47,7		22,1	77,9	
Não	32,8	67,3		15,9	84,1	

AF: atividade física.

Os preditores do impacto no nível de atividade física e no aumento do tempo sentado devido à pandemia da COVID-19 estão descritos na tabela 3. A análise ajustada revela que os adultos que eram mais velhos (p=0,001), inativos (p<0,001) e com sobrepeso (0,005) e doenças crônicas (0,027) tiveram maior impacto no nível de atividade física em decorrência da COVID-19. O aumento do tempo sentado estava associado aos participantes mais velhos (p<0,001), inativos (p=0,002), com doenças crônicas (p=0,001), maior número de dias em isolamento social (p=0,002) e alto nível de escolaridade (p=0,002).

**Tabela 3:** Modelos de regressão logística múltipla para prever o impacto sobre o nível de atividade física e o aumento do tempo sentado devido à COVID-19

Variáveis	β (EP)	RC	IC95%	Valor de p
Impacto no nível de AF^*^				
Idade, anos	-0,013 (0,004)	0,98	0,97-0,99	0,001
Fisicamente ativo, sim como referência	0,790 (0,108)	2,20	1,78-2,72	<0,001
Doenças crônicas, não como referência	0,259 (0,117)	1,29	1,03-1,63	0,027
Sobrepeso, não como referência	0,297 (0,105)	1,34	1,09-1,65	0,005
Impacto sobre o tempo sentado^†^				
Idade, anos	-0,029 (0,005)	0,97	0,96-0,98	<0,001
Fisicamente ativo, sim como referência	0,414 (0,134)	1,51	1,16-1,96	0,002
Doenças crônicas, não como referência	0,504 (0,150)	1,65	1,23-2,22	0,001
Isolamento social, dias	0,013 (0,004)	1,01	1,00-1,02	0,002
Escolaridade, baixa^‡^ como referência	0,629 (0,201)	1,87	1,26-2,78	0,002

^*^ teste de Hosmer-Lemeshow: χ^2^ de 11,231, com p=0,189; ^†^ teste de Hosmer-Lemeshow: χ^2^ de 6,825, com p=0,556; ^‡^ ensino fundamental ou ensino médio. β (EP): coeficiente de regressão (erro padrão); RC: razão de chance; IC95%: intervalo de confiança de 95%; AF: atividade física.

## DISCUSSÃO

Os resultados deste estudo indicam que o isolamento social imposto pela pandemia de COVID-19 levou a uma diminuição do nível de atividade física e ao aumento do comportamento sedentário em adultos brasileiros. A idade mais avançada, a presença de doenças crônicas, a inatividade física e o excesso de peso antes da pandemia de COVID-19 induzem a um maior risco de impacto sobre o nível de atividade física. O aumento do tempo sentado está associado com idade mais avançada, inatividade física prévia, presença de doenças crônicas, maior número de dias no isolamento social e nível de escolaridade mais elevado.

Neste estudo, mais de 50% dos homens e mulheres informaram que a pandemia da COVID-19 reduziu seu nível de atividade física. Curiosamente, essas alterações ocorreram de forma semelhante em todas as faixas etárias. Esses resultados são compatíveis com os relatórios de um monitor de atividade física portátil usado por 30 milhões de pessoas no mundo. O monitor identificou redução de 7% a 38% na contagem de passos durante a semana de 15 de março de 2020 em comparação com o mesmo período do ano anterior.^(7)^

Esse é um dado preocupante, uma vez que a inatividade física e o maior tempo de permanência sentado são preditores independentes de mortalidade por todas as causas e por doenças cardiovasculares. Os efeitos adversos das variáveis inatividade física e comportamento sedentário são múltiplos e estão diretamente relacionados a sarcopenia,^(8)^ maior ocorrência de quedas entre pessoas idosas,^(9,10)^ hipertensão,^(11)^ resistência à insulina e diabetes tipo 2,^(12)^ obesidade,^(13)^ câncer^(14)^ e, consequentemente, maior taxa de mortalidade.^(15,16)^

A inatividade física é a quarta principal causa de morte no mundo e também está associada à redução da expectativa e da qualidade de vida.^(17,18)^ Destaca-se que os custos relacionados à inatividade física, como gastos com saúde e perdas de produtividade, ultrapassaram US$ 67,5 bilhões, em 2013.^(19)^

A deterioração do bem-estar e da qualidade de vida causada pela inatividade física e pelo aumento do comportamento sedentário enfatiza a importância fundamental da atividade física na vida de todos os indivíduos. O aumento do nível de atividade física, como a quantidade de passos, causa melhora significativa da saúde e está inversamente associado ao desenvolvimento de doenças crônicas.^(17-20)^ Diretrizes voltadas para o aumento da atividade física^(21)^ são cruciais para a manutenção da massa muscular e da função neuromuscular, a aptidão cardiorrespiratória e o metabolismo da glicose, especialmente quando circunstâncias inesperadas (como o surto recente de COVID-19) causam uma ampla restrição dos movimentos diários em comparação com a vida normal. Vale ressaltar que a redução do nível de atividade física e o aumento do tempo sentado entre os indivíduos mais velhos, com doenças crônicas ou que já eram inativos antes da pandemia foram ainda maiores.^(22)^

Surpreendentemente, demonstrou-se que os adultos mais velhos, que tinham alguma doença crônica e já eram inativos foram os que mais sofreram com a pandemia, pois apresentaram aumento do tempo sentado e redução do nível de atividade física. Provavelmente, também foram os últimos a deixar o isolamento social. Esses indivíduos são precisamente os que mais precisam de atividade física, principalmente durante o autoisolamento. Esses achados apontam para a necessidade de implementar medidas preventivas de saúde pública que incentivem a prática de atividades físicas em casa durante a pandemia. Tais medidas são especialmente imperativas, porque não se sabe quando essas circunstâncias árduas cessarão. Diversos estudos têm sugerido que a prática de exercícios físicos em casa durante a pandemia pode ser eficaz, embora sejam necessárias mais evidências para determinar os benefícios e as consequências para a saúde em longo prazo.^(23-26)^ O uso das mídias sociais ou de outros recursos on-line especificamente voltados para a atividade física pode ser um método viável para ajudar a aumentar a prática regular de atividades físicas em casa.

Vale destacar que este estudo teve algumas limitações. Seu desenho transversal é uma limitação evidente, pois não permite inferir nenhuma causalidade. Além disso, pesquisas on-line são suscetíveis a vieses de informações e sociais, especialmente quando não há indicadores diretos de atividade física e do comportamento sedentário. Por outro lado, o grande tamanho da amostra e a avaliação oportuna dos comportamentos relacionados à saúde tornam os achados importantes e bastante relevantes.

## CONCLUSÃO

Este estudo demonstrou claramente que idade avançada, presença de doenças crônicas e inatividade física antes do isolamento social tiveram impacto imenso no nível de atividade física, bem como influência prejudicial no aumento do comportamento sedentário durante a pandemia da doença coronavírus 2019.
